# miR-451a and IL18 can differentiate familial Mediterranean fever patients in attack and remission periods: a prospective cross-sectional study

**DOI:** 10.1007/s10067-025-07359-2

**Published:** 2025-02-11

**Authors:** Menderes Yusuf Terzi, Oğuzhan Özcan, Gezmiş Kimyon, Hamza Malik Okuyan, Abdullah Arpacı, Serdar Doğan

**Affiliations:** 1 Department of Medical Biology, Faculty of Medicine, Hatay Mustafa Kemal University, Antakya, Hatay Türkiye; 2 Department of Molecular Biochemistry and Genetics, Graduate School of Health Sciences, Hatay Mustafa Kemal University, Antakya, Hatay Türkiye; 3 Department of Medical Biochemistry, Faculty of Medicine, Hatay Mustafa Kemal University, Antakya, Hatay Türkiye; 4 Department of Internal Medicine, Division of Rheumatology, Faculty of Medicine, Hatay Mustafa Kemal University, Antakya, Hatay Türkiye; 5 Department of Physiotherapy and Rehabilitation, Faculty of Health Sciences, Sakarya University of Applied Sciences, Sakarya, Türkiye

**Keywords:** Familial Mediterranean fever, Inflammation, IL18, M694V mutation, miRNAs

## Abstract

**Objectives:**

Familial Mediterranean fever (FMF) is a multifaceted autoimmune disease and requires a diligent strategical approach considering disease periods and mutation subtypes. We aimed to investigate serum levels of autoimmunity-related cell-free miRNAs and inflammatory and apoptotic markers in FMF patients.

**Methods:**

Sixty FMF patients, of which 30 were in attack (FMF-A) and 30 were in remission (FMF-R) periods, and 25 age-, sex-, and BMI-matched healthy controls were included in our study. The expression levels of miR-26a-5p, miR-146a-5p, miR-155–2-5p, and miR-451a were analyzed with reverse-transcriptase quantitative polymerase chain reaction, and protein levels of interleukin-18 (IL18) and soluble Fas cell surface death receptor (sFAS) were measured with enzyme-linked immunosorbent assay. Serum CRP levels were analyzed by nephelometry, ferritin levels by chemiluminescence, and routine biochemical parameters by spectrophotometry. Correlation analyses were performed to seek potential associations of miRNAs with serum markers and biochemical parameters. Potential biomarkers were tested with receiver operating characteristic analysis.

**Results:**

We observed elevated serum IL18 levels but not sFAS, in FMF patients, particularly during attack period. IL18 demonstrated diagnostic value and was significantly correlated with acute-phase markers namely CRP, fibrinogen, and ferritin. Altered levels of IL18 and miR-451a could distinguish FMF patients in the attack period from the ones in remission. miR-26a-5p, miR-146a-5p, and miR-155–2-5p were downregulated in FMF patients carrying M694V mutations.

**Conclusions:**

These findings suggest that IL18 and specific miRNAs can serve as potential biomarkers for FMF pathogenesis. Discovering promising targets for FMF-related miRNAs using mechanistic strategies will enhance our understanding of FMF disease management and therapy.

**Table:** 

Key Points
• *miR-451a and IL18 can serve as an indicator in distinguishing familial Mediterranean fever patients in attack and remission periods*. • *miR-26a-5p, miR-146a-5p, and miR-155–2-5p were dysregulated in FMF patients carrying M694V mutation*.

**Supplementary Information:**

The online version contains supplementary material available at 10.1007/s10067-025-07359-2.

## Introduction

Familial Mediterranean fever (FMF) is an autosomal recessively inherited autoimmune disease characterized by lifelong periodic fever attacks and serosal inflammation [1]. FMF disease incidence, especially among the people living around the Mediterranean region, is higher than the general population [2]. FMF disease emerges due to a gain of function mutation in the Mediterranean fever innate immunity regulator gene (*MEFV*) comprising 10 exons located at 16 chromosome, which encodes a regulatory protein called pyrin that plays a role in triggering inflammatory responses post-traumatic incidences in the body [3]. Pyrin is commonly expressed in innate immune cells, e.g., macrophages, NK cells, and granulocytes, and is involved in several physiological and pathological events linked to inflammation and survival pathways such as inflammasome formation, caspase 1 (CASP1) activation, the regulation of pro-interleukin 1β (pro-IL1β) activation, and pyroptosis [4]. In this context, mutant pyrin can lead to deregulations in the secretion of IL1β and IL18 cytokines through inflammasomes [5, 6]. The fact that the FMF patients or heterozygous carriers with the same mutation genotype have various symptoms at different severity levels makes the researchers consider the possibility that other accompanying mutant genes and/or epigenetic and environmental factors can play a role in the FMF disease onset and symptomatic heterogeneity [7]. In general, the mutation subtype in the pyrin-encoding *MEFV* gene is not the only indicator regarding the FMF disease onset, which makes FMF a more complicated disease in terms of diagnosis, disease course, and therapy [8].

MicroRNAs (miRNAs) are 19–22 base-long oligonucleotides involved in post-transcriptional regulation as one of the epigenetic mechanisms [9]. miRNAs have been implicated in several disorders such as autoimmune diseases via modulating vital pathways, e.g., cell proliferation, apoptosis, and inflammation. Particularly, circulating cell-fee miRNAs in serum/plasma have a promising potential to be utilized as a therapeutic agent/target or diagnostic marker in several pathophysiological conditions [10, 11].

In a clinical study performed using peripheral blood mononuclear cells (PBMCs) of FMF patients, the levels of 10 miRNAs including miR-451a were reported to be dysregulated in homozygous M694V mutation-carrying patients compared to control subjects [12]. In other clinical studies conducted with FMF patients, circulating miRNAs including miR-26a-5p, miR-146a, and miR-155–2-5p were found to be deregulated [13–15]. Although the serum levels of certain cytokines, i.e., IL6, IL17, and IL18, have been reported to increase in FMF patients [16, 17], there is no common biomarker for FMF diagnosis other than DNA sequencing as the gold standard method [18]. Nonetheless, in autoimmunity-related disorders, circulating apoptotic molecules such as sFAS, sFASL, and sTRAIL in circulation were highlighted to be utilized for diagnostic and prognostic purposes [19, 20].

In this context, we first aimed in the present study to analyze serum levels of certain cell-free miRNAs (miR-26a-5p, miR-146a-5p, miR-155–2-5p, and miR-451a) and IL18 and sFAS protein levels in FMF patients. Second, we evaluated the relationship of cell-free miRNA expressions with several disease genotypes, biochemical parameters, and different periods of the disease.

## Patients and methods

### Ethical statement

**Fig. 1:**
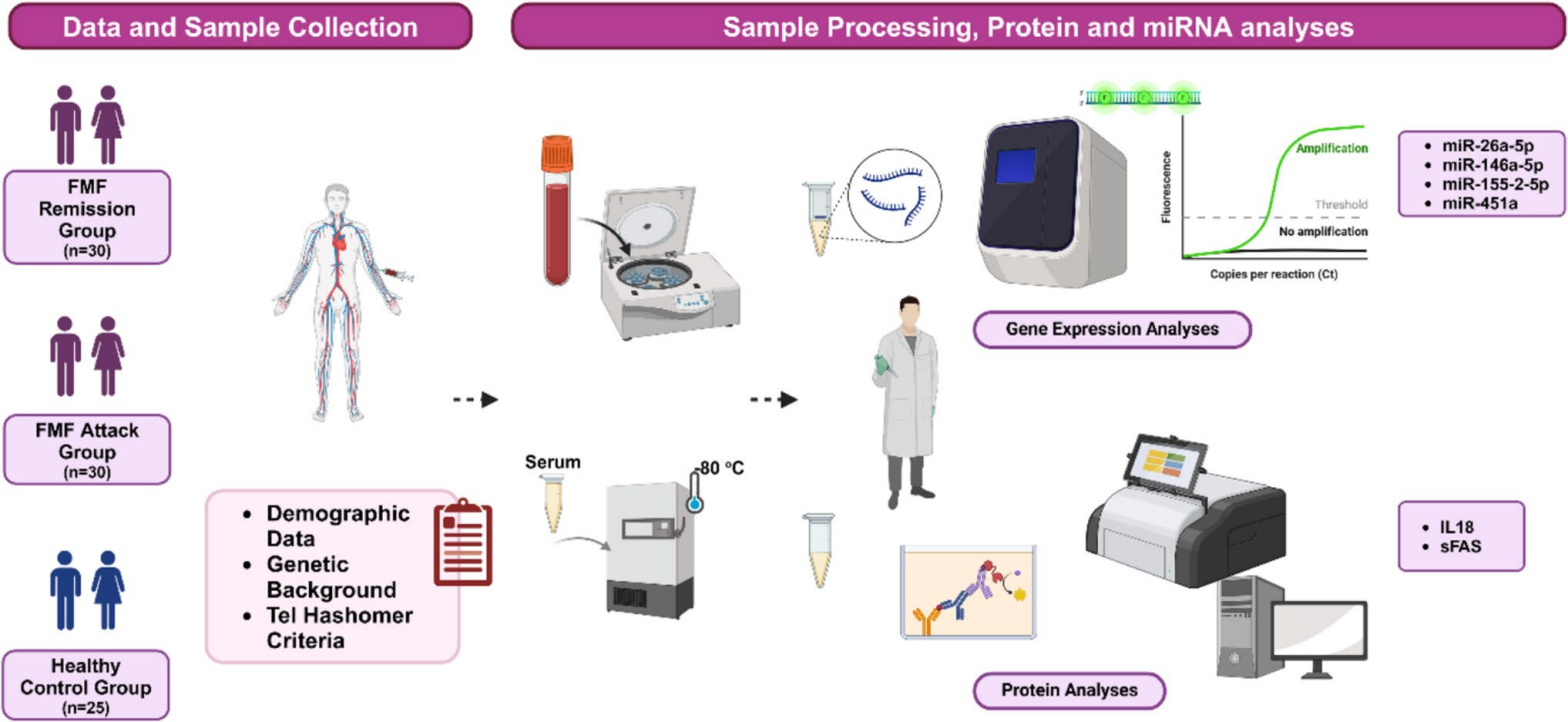
The schematic design of the study. FMF -A, -R, familial Mediterranean fever -attack, -remission

This prospective cross-sectional study was conducted after approval by the Clinical Research Ethics Committee of Hatay Mustafa Kemal University (Approval No: 2020/73), under the terms of the Helsinki Declaration’s protocol on working with human subjects. The study design was summarized in Fig. 1. Sixty FMF patients, of which 30 were in attack (FMF-A) and 30 were in remission periods (FMF-R), and 25 sex-, age-, and body mass index (BMI)-matched healthy controls without any familial FMF history were included in the present study after the signed informed consents were obtained from each participant. The FMF patients were diagnosed by a rheumatologist based on the Tel Hashomer criteria [21]. All patients were under colchicine treatment. The total number of participants for the study was calculated with power analysis (G-power v.3.1.9.6). The exclusion criteria of the study were as follows: people < 18 years, pregnant or lactating women, patients in intensive care unit, patients who have acute or chronic infectious diseases, were under glucocorticoid and/or immunomodulant/immunosuppressive medication within last 3 months, have other auto-immune disorders, and bear FMF symptoms without any established FMF mutations.

### Blood sample collection

The sera samples were harvested after venipuncture peripheral blood collection from all participants into test tubes without anticoagulant reagent and centrifugation at 2000 × g, 4 °C for 15 min. All the sera samples were aliquoted and stored in RNase-free tubes at − 80 °C until further experiments.

### Analysis of clinical parameters

The demographic features of the subjects (age, sex, BMI) together with their physical and clinical symptoms (peritonitis, fever, arthritis, others, i.e., muscle, chest, and joint pains, rash, MEFV gene mutation type (homozygous, heterozygous, other mutations), disease duration) were recorded by a rheumatology expert. Serum levels of glucose, albumin, ALT, AST, CK, BUN, creatinine, and SAA were measured by spectrophotometry, while urine microalbumin was quantified by immunoturbidimetry on an autoanalyzer (Siemens Advia 2400, Japan). Serum CRP levels were determined by nephelometry (Siemens BN II, Germany) and ferritin by chemiluminescence method (Siemens XPT, USA). Hemogram parameters were assessed with an automated blood count device (Mindray BC6800, China), and fibrinogen levels were measured using an autoanalyzer (Stago Compact Max, Netherlands).

### Protein assay with enzyme-linked immunosorbent assay

Enzyme-linked immunosorbent assay (ELISA) was used for spectrophotometric measurement of IL18 and sFAS proteins in the sera of all participants (Multiskan GO, Thermo Fisher Scientific) by following the commercial kits’ protocols. The intra- and inter-assay coefficients of variation (CV) of ELISA kits were as follows: IL18 (BMS267-2, Invitrogen); CVs for intra and inter-assay were 6.5% and 8.1%, respectively. sFAS (BMS245, Invitrogen); CVs for intra and inter-assay were 4.5% and 3.1%, respectively.

### Quantitative analysis of relative miRNA expressions

The mRNA expression levels of mature miRNAs were analyzed using the reverse transcription-quantitative PCR (qRT-PCR) method as described previously [22]. Briefly, total RNA isolation from sera samples was performed using miRNeasy Serum/Plasma Kit (Qiagen, Hilden, Germany). Further, cDNA library synthesis (miRCURY LNA miRNA RT Kit, Qiagen, Hilden, Germany) was carried out using an equal volume of eluted RNA samples (2 µL) since the total RNA concentrations of the sera samples were not enough to be quantified with conventional spectrophotometric methods. The reaction conditions for the cDNA synthesis were as follows: 60 min at 42 °C, 5 min at 95 °C, and 4 °C for short-term storage. The cDNA samples were pre-diluted for further qPCR reactions that were performed using miRCURY LNA SYBR Green PCR Kit (Qiagen, Hilden, Germany) in Rotor-Gene Q real-time thermal cycler (Qiagen, Hilden, Germany). Since there is no common normalization method of the raw qPCR data for the bodily fluid samples (serum/plasma, milk, saliva, urine, etc.), circulating serum miR-191-5p expression levels were utilized for normalization as previously suggested [18]. miRCURY LNA miRNA PCR Assays (Qiagen, Hilden, Germany) were used as pre-designed primers that are specific for our target miRNA genes which were as follows: hsa-miR-26a-5p (YP00206023), hsa-miR-146a-5p (YP00204688), hsa-miR-155–2-5p (YP02119311), hsa-miR-451a (YP02119305), and housekeeping hsa-miR-191-5p (YP00204306). The target miRNAs were selected from the previous studies that are related to FMF disease and by using the Human microRNA Disease Database [23]. qPCR reaction conditions were as follows: one-step cycle at 95 °C for 2 min, 40 cycles of 10 s at 95 °C and 60 s at 56 °C, and final melting-curve analysis between 60 and 95 °C. The amplicons that are specific for the target genes were confirmed after monitoring melting curves (Supplementary Figs. 1–5). We used the 2^−Δct^ method to calculate relative gene expression levels.

### Statistical analysis

We first utilized the Shapiro–Wilk test to evaluate the Gaussian distribution of the data. Then, we compared all groups using the Kruskal–Wallis H test, while we used the Mann–Whitney U test to compare two individual groups. We performed the Spearman’s rank correlation test to analyze the relationship of the candidate miRNAs with other parameters. The Wilcoxon signed-rank test was used to compare paired samples’ miRNA levels. We carried out the receiver operating characteristic (ROC) analyses to evaluate the diagnostic value of the candidate markers. The continuous values were presented as mean ± SEM or median (min–max), while the categorical values were shown as percentages. We considered *p*-values less than 0.05 as statistically significant.

## Results

### Demographic and clinical parameters

The demographics, major clinical parameters, and ELISA results of FMF patients and control subjects are summarized in Table 1. Briefly, there was no significant difference between patient and control groups concerning gender, age, and BMI values (*p* > 0.05). In the FMF-R group, 4 patients had the following comorbidities; ankylosing spondylitis, Behçet’s disease, type-2 diabetes mellitus, and nephrotic syndrome, while in the FMF-A group, only one patient had comorbidity of cardiac valve disease. Seventy-one percent of the FMF patients included in the present study had a heterozygous mutation, while the rest 29% had a homozygous mutation. The number of homozygous M694V mutation-carrying FMF patients (22%), which bear the most severe FMF symptoms, was less than the heterozygous M694V (32%) and other mutation (47%) carrying patients. The disease duration of all the FMF patients varied between 2 and 40 years, and 40% of all the FMF patients exhibited at least a common FMF symptom, of these ~ 92% belonged to the FMF-A group. Forty-five percent of the FMF patients had a family history of FMF disease. CRP and SAA levels were also analyzed in FMF patients, and we found a significant increment in CRP levels in FMF-A, FMF-R, and all the FMF patients compared to the control (*p* < 0.05). In addition, the CRP level in FMF-A was also higher than the one in the FMF-R group (*p* < 0.05). As to SAA, there was a significant difference between FMF-A and FMF-R groups (*p* < 0.05).

**Table 1:** Demographic and clinical parameters with ELISA results of control and FMF patients

	Control	FMF-A	FMF-R	FMF (all)	*p*-value
Age (mean ± SEM)	31.9 ± 7.2	31.0 ± 9.1	32.1 ± 12.3	31.6 ± 10.8	0.899
Gender *n* (%) Female Male	12 (48.0%) 13 (52.0%)	15 (50.0%) 15 (50.0%)	19 (36.7%) 11 (63.3%)	34 (56.7%) 26 (43.3%)	0.448
BMI (mean ± SEM)	23.4 ± 3.01	23.6 ± 3.2	23.4 ± 2.8	23.5 ± 3.0	0.938
Comorbidity *n* (%)					
Ankylosing spondylitis		1 (3.3%)	-	1 (1.7%)	
Behçet’s disease		1 (3.3%)	-	1 (1.7%)	
Type-2 DM	-	1 (3.3%)	-	1 (1.7%)	-
Nephrotic syndrome		1 (3.3%)	-	1 (1.7%)	
Cardiac valve disease			1 (3.3%)	1 (1.7%)	
Homozygous *n* (%) Heterozygous *n* (%)	-	6 (20.0%) 24 (80.0%)	11 (39.3%) 17 (60.7%)	17 (29.3%) 41 (70.7%)	0.107
Mutations *n* (%)					
Homo M694V Hetero M694V Other mutations	-	5 (16.7%) 12 (40.0%) 13 (43.3%)	8 (26.7%) 7 (23.3%) 15 (50.0%)	13 (21.7%) 19 (31.7%) 28 (46.7%)	0.341
Disease duration (mean ± SEM)	-	16.6 ± 9.1	15.0 ± 9.0	15.8 ± 9.0	0.519
Symptoms *n* (%)					
Peritonitis Fever Arthritis Peritonitis + fever Others	-	11 (36.7%) 1 (3.3%) 2 (6.7%) 6 (20.0%) 2 (6.7%)	- - 2 (6.7%) - -	11 (18.3%) 1 (1.7%) 4 (6.7%) 6 (10.0%) 2 (3.3%)	
CRP, mg/L median (min–max)	1.0 (0.1–4.8)	15.8 (3.1–214.0)	3.1 (2.1–15.2)	5.2 (2.1–214.0)	** < 0.0001**^**a**^ **0.0061**^**b**^ ** < 0.0001**^**c**^ ** < 0.0001**^**d**^
SAA, mg/L median (min–max)	-	50.2 (1.0–300.0)	7.4 (2.4–28.8)	10.0 (1.0–300.0)	**0.0008**^**d**^
IL18, pg/mL median (min–max)	183.9 (23.7–515.7)	563.9 (22.2–5928)	252.4 (23.1–2047)	301.5 (22.2–5928)	**0.002**^**a**^ **0.032**^**c**^
sFAS, pg/mL median (min–max)	101.9 (59.1–304.4)	92.4 (64.5–254.4)	90.0 (40.1–251.5)	91.9 (40.1–254.4)	> 0.05

a: Comparison between FMF-A and control groups. b: Comparison between FMF-R and control groups. c: Comparison between FMF (all) and control groups. d: Comparison between FMF-A and FMF-R groups. *p *< 0.05 denotes statistical significance (typed in bold) *FMF-A, -R*, familial Mediterranean fever-attack, -remission; *DM*, diabetes mellitus; *SAA*, serum amyloid A; *CRP*, C-reactive protein; *BMI*, body mass index; *SEM*, standard error of mean; *IL18*, interleukin-18; *sFAS*, soluble Fas cell surface death receptor

### Elevated serum IL18 level in FMF patients despite steady sFAS level

We performed ELISA analyses to determine the levels of certain inflammatory and apoptotic markers in the FMF patients (Table 1). Serum IL18 levels in all FMF patients increased notably compared to the control group (*p* < 0.05). Additionally, the difference between FMF-A and control group regarding IL18 levels was more substantial (*p* = 0.002), while the IL18 level in the FMF-R group did not show a significant difference compared to the control group (*p* > 0.05). There was no statistical difference in serum sFAS levels among the groups (*p* > 0.05).

### Differential expression of miR-451a in FMF-A and FMF-R patients

**Fig. 2:**
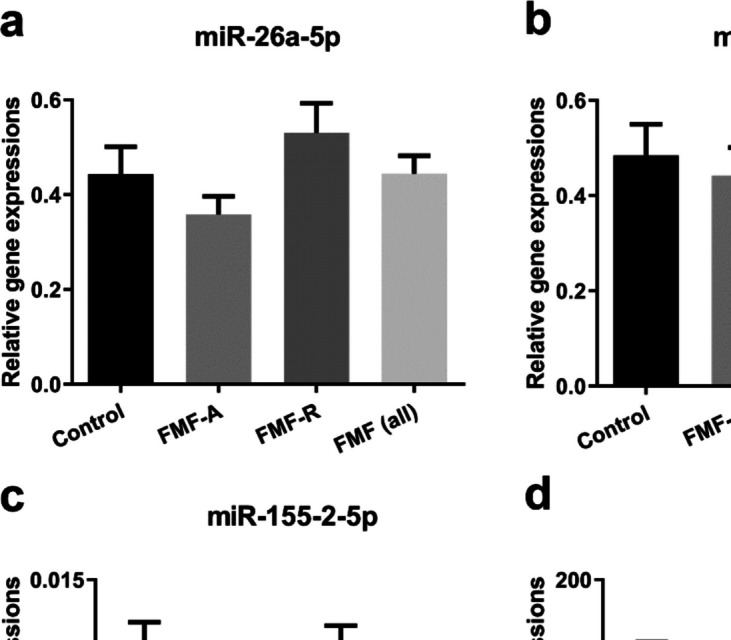
Relative expression levels of miRNAs; **a** miR-26a-5p, **b** miR-146a-5p, **c** miR-155–2-5p, and **d** miR-451a in control and FMF patients in attack (FMF-A) or remission (FMF-R) periods. Relative expressions were calculated with 2^−ΔCt^. Data were presented as mean ± SEM, *p* < 0.05 denotes statistical significance, n_control_ = 25, n_FMF-A,-R_ = 30, n_FMF (all)_ = 60. FMF -A, -R, familial Mediterranean fever -attack, -remission; SEM, standard error of mean

FMF disease-related target miRNAs in the current study were analyzed using the RT-qPCR method (Fig. 2), and we found no significant difference in miR-26a-5p, miR-146a-5p, miR-155–2-5p, and miR-451a levels between all the FMF patients and the control group (*p* > 0.05). However, the miR-451a expression level in the FMF-A group was lower than the one in the FMF-R group (Fig. 2d, *p* > 0.05).

### Altered expression of miRNAs in FMF patients depending on mutation subtype

**Fig. 3:**
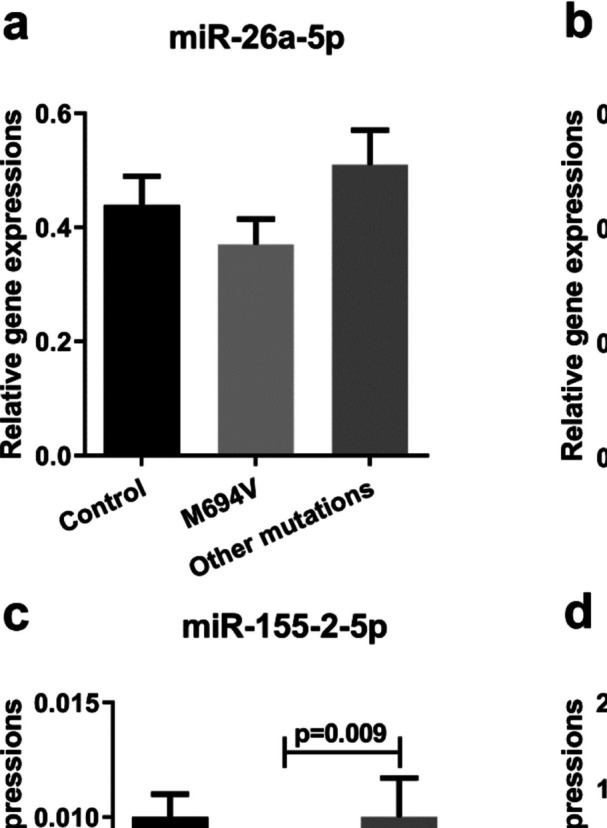
Relative expression levels of miRNAs; **a** miR-26a-5p, **b** miR-146a-5p, **c** miR-155–2-5p, and **d** miR-451a in control and FMF patients who have at least one M694V mutation and other mutations. Relative expressions were calculated with 2.^−ΔCt^. Data were presented as mean ± SEM, *p* < 0.05 denotes statistical significance, n_control_ = 25, n_M694V_ = 32, n_Other mutations_ = 28

**Fig. 4:**
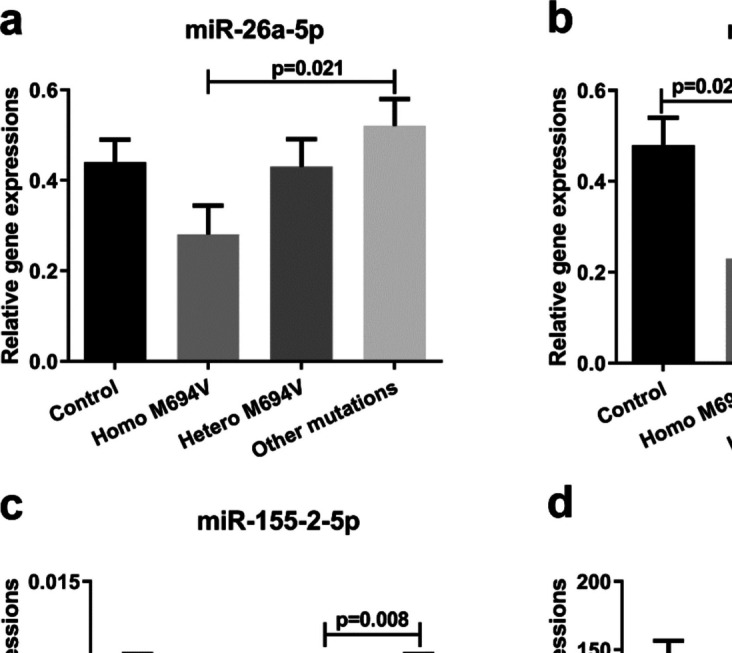
Relative expression levels of miRNAs; **a** miR-26a-5p, **b** miR-146a-5p, **c** miR-155–2-5p, and **d** miR-451a in control and FMF patients who have homozygous and heterozygous M694V mutations and other mutations. Relative expressions were calculated with 2.^−ΔCt^. Data were presented as mean ± SEM, *p* < 0.05 denotes statistical significance, n_control_ = 25, n_HomoM694V_ = 14, n_HeteroM694V_ = 18, n_Other mutations_ = 28

Further analyses conducted among FMF patients carrying different FMF mutation subtypes revealed that miR-146a-5p and miR-155–2-5p levels in FMF patients carrying at least one M694V mutation were significantly lower than the ones in FMF patients with other mutations (Fig. 3b, c, *p* < 0.05). Moreover, the miRNA levels were also analyzed after subclassifying the FMF mutations under homozygous and heterozygous M694V and other mutations. The FMF patients with homozygous M694V mutation had significantly lower miR-26a-5p and miR-146a-5p levels compared to the ones carrying other mutations (Fig. 4a, b, *p* < 0.05). Besides, FMF patients with homozygous M694V mutation had also lower miR-146a-5p levels compared to healthy control subjects (Fig. 4b, *p* < 0.05). Additionally, miR-155–2 levels in FMF patients with heterozygous M694V mutation significantly decreased compared to the ones with other mutations (Fig. 4c, *p* < 0.05). However, miR-451a levels did not show any significant alteration depending on the FMF mutation subtype (Figs. 2 and 3, *p* > 0.05).

### Steady miRNA levels between attack and remission periods of the same FMF patients

**Fig. 5:**
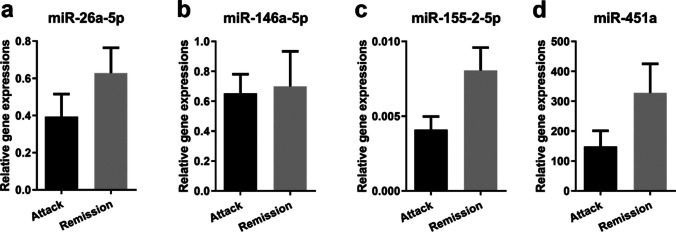
Relative expression levels of miRNAs; **a** miR-26a-5p, **b** miR-146a-5p, **c** miR-155–2-5p, and **d** miR-451a in the same FMF patients who were in attack and remission periods. Relative expressions were calculated with 2.^−ΔCt^. Data were presented as mean ± SEM, *n* = 6

Six FMF patients were further analyzed during their attack and remission periods, and we found no significant change in the levels of target miRNAs between the two disease periods (Fig. 5, *p* > 0.05).

### Correlation analysis

The correlation analyses of the miRNAs and ELISA markers with the whole blood counts and other biochemical parameters were summarized in Table 2. We demonstrated that all the target miRNAs positively correlated with each other (*p* < 0.05), while IL18 and sFAS levels did not show any significant correlation with the target miRNAs or with each other (*p* > 0.05). Besides, IL18 had significant correlations with fibrinogen, AST, ferritin, and CRP levels (*p* < 0.05). miR-26a had significant correlations with WBC, RBC, ALT, AST, and glucose; miR-146a with CRE, BUN, and CK; mir-155–2 with basophil and AST; miR-451a with hemoglobin, AST, and ALT levels (*p* < 0.05).

**Table 2:**
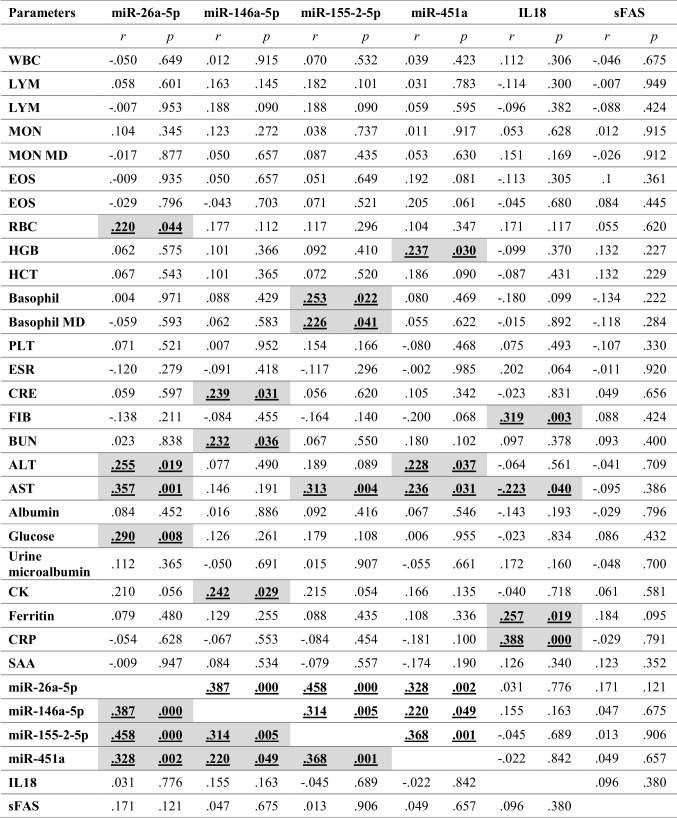
Correlation analyses

*r*, correlation coefficient; *p* < 0.05 denotes statistical significance (shaded cells); *WBC*, white blood cell; *LYM MD*, lymphocyte mean density; *MON*, monocyte; *EOS*, eosinophil; *RBC*, red blood cell; *HGB*, hemoglobin; *HCT*, hematocrit; *PLT*, platelet; *ESR*, erythrocyte sedimentation rate; *CRE*, creatinine; *FIB*, fibrinogen, *BUN*, blood urea nitrogen; *ALT*, alanine transaminase; *AST*, aspartate aminotransferase; *CK*, creatine kinase; *CRP*, C-reactive protein; *SAA*, serum amyloid A; *IL18*, interleukin-18; *sFAS*, soluble Fas cell surface death receptor

### ROC analyses and diagnostic value of serum IL18 level in FMF patients

**Fig. 6:**
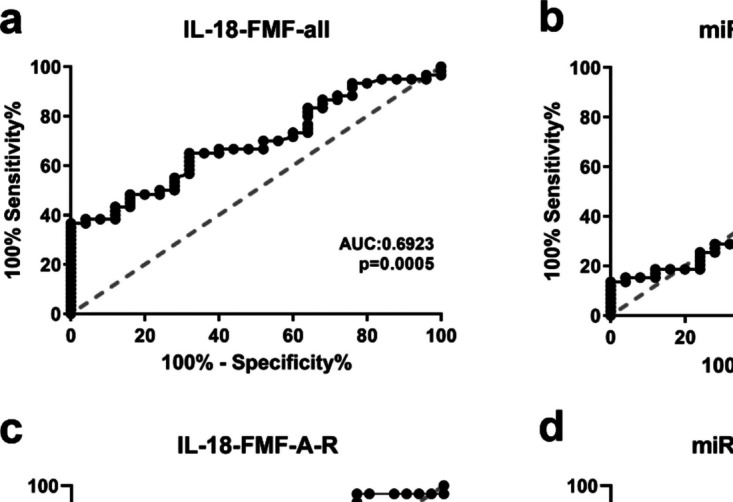
ROC curve analyses demonstrating the potential diagnostic values of **a, c** IL18 and miR-451a (**b, d**) for all FMF patients vs. control and FMF-A vs. FMF-R patients. ROC analysis details were summarized in Supplementary Table 1. *p* < 0.05 denotes statistical significance, ROC: Receiver operating characteristic, AUC: Area under the curve, FMF -A, -R, familial Mediterranean fever -attack, -remission

ROC analysis was performed to evaluate the diagnostic value of IL18 and miR-451a as depicted in Fig. 6, and its details were summarized in Supplementary Table 1. We observed that IL18 exhibited a significantly higher area under the curve (AUC) value (Fig. 6a, AUC: 0.69, *p* < 0.05) among all FMF patients, meaning that IL18 can be used as a distinctive diagnostic indicator for FMF disease. miR-451a did not have a diagnostic factor in distinguishing all the FMF patients from healthy controls (Fig. 6b, AUC: 0.50, *p* > 0.05). On the other hand, ROC analyses for IL18 (Fig. 6c, AUC: 0.69, *p* < 0.05) and miR-451a (Fig. 6d, AUC: 0.70, *p* < 0.05) exhibited significant distinctive power between the FMF patients in the attack and remission periods. IL18 expressed higher AUC values when the analysis was performed solely with FMF-A patients and healthy controls (AUC: 0.78, data not shown).

## Discussion

FMF is a complex genetically inherited disease accompanied with several symptoms at different levels depending on the mutation subtype, epigenetics, and other environmental factors [24]. In the present study, we aimed to investigate the serum miRNA levels of autoimmune disease-related miRNAs and their potential association with inflammatory marker IL18 and apoptotic marker sFAS in FMF patients. We found altered levels of miR-451a and IL18 which can be utilized to differentiate FMF patients in attack and remission periods. Besides, miR-26a-56, miR-146a-5p, and miR-155–2-5p levels were deregulated in FMF patients carrying FMF mutation subtypes, i.e., homozygous and heterozygous M694V and other mutations. We also analyzed the levels of target miRNAs during the attack and remission periods of 6 FMF patients; however, we found no marked change due to a restricted number of data for two disease periods of the same patients.

Kiraz et al. demonstrated that serum sFAS levels were increased in attack-free FMF patients without amyloidosis, while FMF patients with decreased sFAS levels had amyloidosis [25]. The same group claimed that sFAS can behave like a FAS antagonist and inhibit FAS-promoted apoptosis and amyloidosis. In our study group, none of the FMF patients developed amyloidosis either in attack or remission periods, and we found no significant difference among study groups regarding serum sFAS levels. Therefore, steady sFAS levels during attack and remission periods of FMF patients cannot be implicated in amyloidosis development. A previous study conducted with FMF patients in the attack period showed that the serum levels of inflammatory cytokines IL6, IL17, and IL18 were closely associated with the FMF disease [16]. In other similar studies, IL18 levels were reported to increase in FMF patients compared to the control subjects [26, 27]. In line with these findings, we demonstrated increased IL18 levels in FMF patients compared to the control group. Further, our ROC analyses also revealed that IL18 had diagnostic strength in distinguishing FMF patients in the attack and remission periods. Previous studies have also investigated the clinical value of IL18 in various diseases [28] and reported that IL18 elevated in IgA nephropathy, systemic lupus erythematosus, and fatal cardiovascular diseases [29–31].

The serum levels of FMF patients in the attack and remission period were measured with RT-qPCR, and we found no significant change in the serum levels of miR-26a-56, miR-146a-5p, miR-155–2-5p, and miR-451a between all FMF patients and control groups. Hortu et al. analyzed the expression levels of 15 miRNAs in whole blood samples of FMF patients and reported that miR-26a-56, miR-146a-5p, and miR-155–2-5p levels decreased in FMF patients compared to control [14]. On the other hand, after comparing the miRNA levels between the FMF patients in the attack and remission groups, we observed that the miR-451a level in FMF-A was markedly downregulated compared to the one in FMF-R, whereas there was no significant alteration in miR-26a-5p, miR-146a, and miR-155–2-5p levels. Demir et al. reported that plasma miR-155–2-5p levels decreased in the remission period of FMF patients compared to control, while miR-451a levels did not show a significant change [13]. Hortu et al. also compared miRNA levels between remission and attack periods in pediatric FMF patients and asserted that miR-26a-5p and miR-146a levels in whole blood samples were markedly decreased compared to control [14]. In another study by Karpuzoglu et al., the plasma levels of certain miRNAs were analyzed in pediatric FMF patients, and they revealed that 19 miRNAs including miR-26a-5p and miR-146a-5p were downregulated compared to control [15]. The potential factors leading to these contradictory findings with our results can arise from the difference in analyzed material, e.g., plasma, serum, whole blood, or PBMCs as well as the altered ages of study populations.

In a clinical study, the serum miRNA levels of FMF patients were measured with the microarray method, and it was suggested that some miRNAs can be used to differentiate sub-FMF mutations [32]. We demonstrated that FMF patients with M694V mutations exhibited lower levels of miR-146a and miR-155–2 compared to the ones with other mutation subtypes. Additionally, miR-26a-5p and miR-451a levels in homozygous M694V-carrying patients did not show a notable change compared to the control group and FMF patients with other mutations. However, in a study conducted with FMF patients carrying homozygous M694V mutation, miR-451a level was demonstrated to be upregulated in PBMC compared to healthy controls [12]. The fact that our study was performed with cell-free circulating miRNAs could bring about this contradiction. miR-451a was previously reported to be implicated in inflammatory pathways via upregulating in rheumatoid arthritis and systemic lupus erythematosus (SLE) [33] and downregulating in influenza-infected dendritic cells which led to the increased secretion of inflammatory cytokines, e.g., IL6 and TNFα [34]. Another clinical study conducted with ankylosing spondylitis patients reported that miRNA-451a was significantly downregulated and demonstrated high power as a diagnostic and prognostic marker [35]. Our ROC analyses showed that miR-451a had strength in distinguishing FMF patients in the attack and remission periods. In this context, we assume that altered expression of miR-451a in FMF patients during the attack period can be linked to elevated IL18 levels even though direct evidence is currently lacking. In the present study, FMF patients in the attack period were classified based on conventional method including the presence of typical symptoms, namely, fever, serositis, and/or erysipelas-like erythema along with elevated acute phase reactants (e.g., CRP and SAA) and relief of the symptoms after attack period. The FMF patients in the remission group were presumed to have no attack. However, some acute phase parameters like CRP during remission can still remain at higher levels compared to healthy controls. Therefore, the combination of classical diagnostic parameters together with novel markers such as miR-451a and IL18 levels, could enhance the accuracy of distinguishing between FMF subtypes and/or FMF patients in different phases. A previous clinical study has reported systemic immune-inflammatory index as a valuable clinical indicator to distinguish FMF patients bearing sacroiliitis [36].

We performed further analyses among the FMF patients with homozygous and heterozygous M694V mutations by comparing them with other FMF mutation subtypes. miR-155–2 levels in heterozygous patients were lower than the patients with other mutations. Besides, miR-26a-5p and miR-146a levels were markedly downregulated in FMF patients with homozygous M694V mutation compared to patients with other mutations. Furthermore, miR-146a was substantially downregulated in FMF patients with homozygous M694V mutation compared to the control group. An in vitro study conducted with mouse macrophages claimed that miR-146a mimics could suppress the expression of IL18, while miR-146a inhibitors notably upregulated IL18 expression [37]. Our results along with the literature findings corroborate the association of elevated serum IL18 levels with suppressed expression of miR-146a in FMF patients with homozygous M694V mutation. The circulating miRNAs have been known to possess diagnostic, prognostic, and therapeutic potentials in the management of several diseases such as cancer, neurodegenerative, and autoimmune disorders [38]. miR-155 has been previously shown to act as an inflammatory modulator in experimental and clinical arthritis [39]. In a clinical study performed with MS patients, serum levels of miR-26a, miR-34a, and miR-146a were increased together with IL17 levels. In the same study, miR-155 level did not show a marked alteration despite a significant negative correlation with IL17. An in vitro model of psoriasis demonstrated that miR-155-5p acts as a proinflammatory factor by exacerbating inflammatory response via the IRF2BP2/KLF2/NF-κB pathway [40]. Furthermore, miR-155 inhibition was demonstrated to alleviate SLE symptoms by preventing regulatory T-cell (Tregs) impairment through the suppressor of cytokine signaling 1 protein in an in vivo mouse model [41]. We speculate that the altered expression of miR-155–2-5p in FMF patients carrying M694V mutation may be linked to the disease severity by modulating inflammation regulatory machinery. miR-146a is known as a key regulatory player of the NF-κB pathway, and it was reported that the knock-down of miR-146a resulted in autoimmune disease and chronic inflammation in mice due to continual NF-κB activation [42]. In a clinical study with SLE patients, miR-146a was implicated in disease pathogenesis by interfering type-1 interferon pathway [43]. Moreover, miR-146a was also shown to alleviate OA-related joint pain in mice [44]. Together with these findings, our results may suggest that suppressed serum levels of miR-146a in M694V mutation-bearing FMF patients can worsen the disease symptoms compared to FMF patients with other mutations. In a bioinformatics study, miR-26a has been detected as a key hub player among autoimmune-related genes in the prediction of moyamoya disease [45]. Previous studies seeking therapeutic targets against experimental autoimmune encephalomyelitis reported that overexpression of miR-26a [46] and miR-146a [47] and suppression of miR-155 [48] could reduce disease symptoms. In a meta-analysis seeking the expression profile of miRNAs linked to autoimmune diseases, several miRNAs including miR-26a and miR-155 have been detected as differentially expressed in more than one disease [49]. In a clinical study, miR-26a, miR-146a, and miR-155 were shown to be differentially expressed in CD4 T and B lymphocytes of the patients with primary Sjögren’s syndrome. An in vivo autoimmune type-1 diabetes mellitus model demonstrated that miR-26a prolonged the normoglycemic time by suppressing autoreactive T-cells and propagating Tregs [50].

## Conclusion

In conclusion, we demonstrated that serum IL18 levels, but not sFAS, were markedly elevated in FMF patients, particularly during attack periods, and correlated with key clinical parameters such as CRP levels. The differential expression of IL18 and miR-451a could distinguish FMF patients in attack and remission periods. miR-26a-5p, miR-146a-5p, and miR-155–2-5p were dysregulated in patients carrying different FMF mutation subtypes. This study provides preliminary insights that have the potential to influence diagnostic and therapeutic strategies. In the diagnostic realm, the precision of distinguishing FMF subtypes or phases may be enhanced by miR-451a and IL18 levels. Therapeutically, the molecular differences have the potential to facilitate the development of personalized treatments, such as those that target IL18 involved pathways or targeting relevant miRNAs in severe FMF cases. Considering FMF as a complex disease, identifying the potential targets of these miRNAs through in vitro and in vivo mechanistic studies is warranted to enhance our understanding of FMF disease management and therapy.

## Limitations of the study

The current study offers some crucial information regarding the involvement of miRNAs in FMF patients; however, our research has certain limitations. The cross-sectional design of the study does not allow for the assessment of changes over time or causality. The study’s sample size is relatively small. The effect of the drug therapy received by the FMF patients on the miRNA expressions and protein levels is unclear. The lack of genetic homogeneity in the study group and other confounder effects, e.g., smoking or drinking habits, can substantially affect the results. Our study was predominantly designed and powered to assess differences in miRNA levels, as this was the primary endpoint of interest. Although we also investigated IL18 levels as a secondary focus, the sample size may not have been sufficient to identify the subtle differences in IL18 expression. Consequently, it is imperative to exercise caution when interpreting the results of the study on miRNAs and IL18, and additional research with larger cohorts is necessary to verify these findings.

## Supplementary Information

Below is the link to the electronic supplementary material.

Supplementary file1 (DOCX 1.44 MB)

Supplementary file2 (DOCX 13.7 KB)

## Data Availability

The datasets generated during and/or analyzed during the current study are available from the corresponding author on reasonable request.
