# Supplementary material for: miR-451a and IL18 can differentiate familial Mediterranean fever patients in attack and remission periods: a prospective cross-sectional study
Source: Clin Rheumatol. 2025 Feb 11;44(4):1691–704. doi: 10.1007/s10067-025-07359-2 (PMC11993460; doi:10.1007/s10067-025-07359-2)
Supplement: Supplementary file 1 — Supplementary file1 (DOCX 1.44 MB) [file 10067_2025_7359_MOESM1_ESM.docx]

**Supplementary Figures**

**Supplementary Fig. 1**


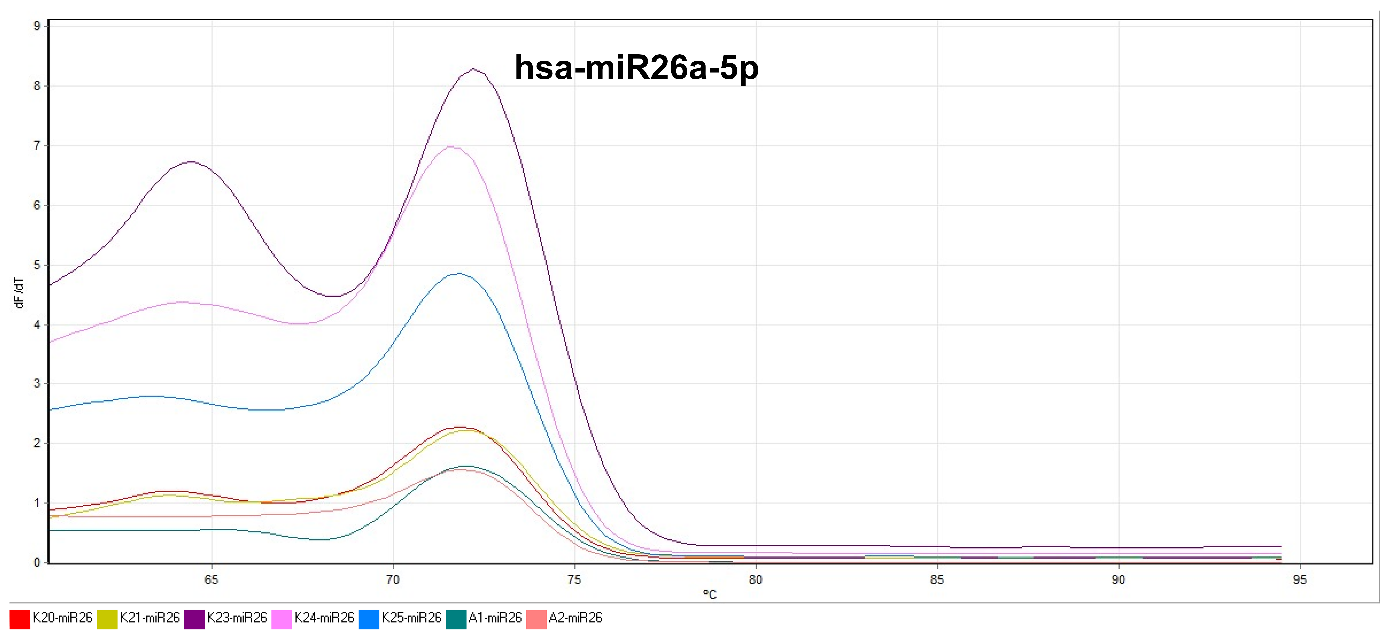


**Supplementary Fig. 1** Melting curve analysis of has-miR26a-5p. Reaction condition: 1°C rise between 60-95°C

**Supplementary Fig. 2**


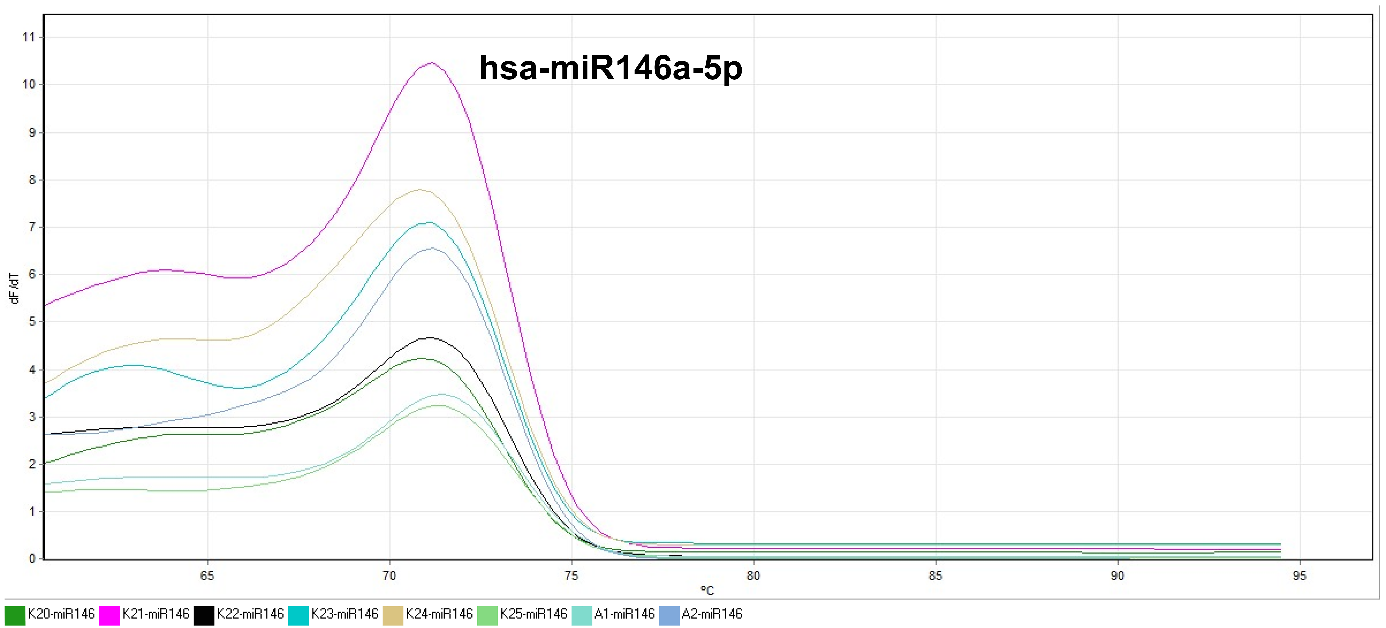


**Supplementary Fig. 2** Melting curve analysis of has-miR146a-5p. Reaction condition: 1°C rise between 60-95°C

**Supplementary Fig. 3**


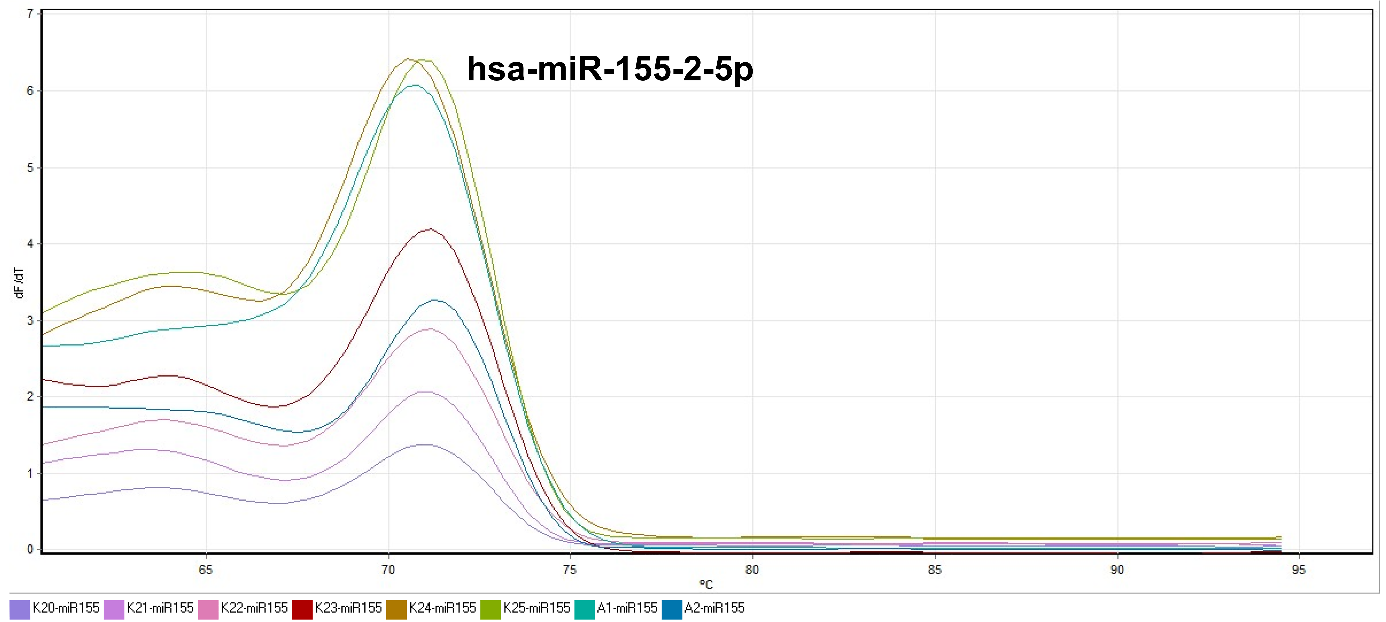


**Supplementary Fig. 3** Melting curve analysis of has-miR155-2-5p. Reaction condition: 1°C rise between 60-95°C

**Supplementary Fig. 4**


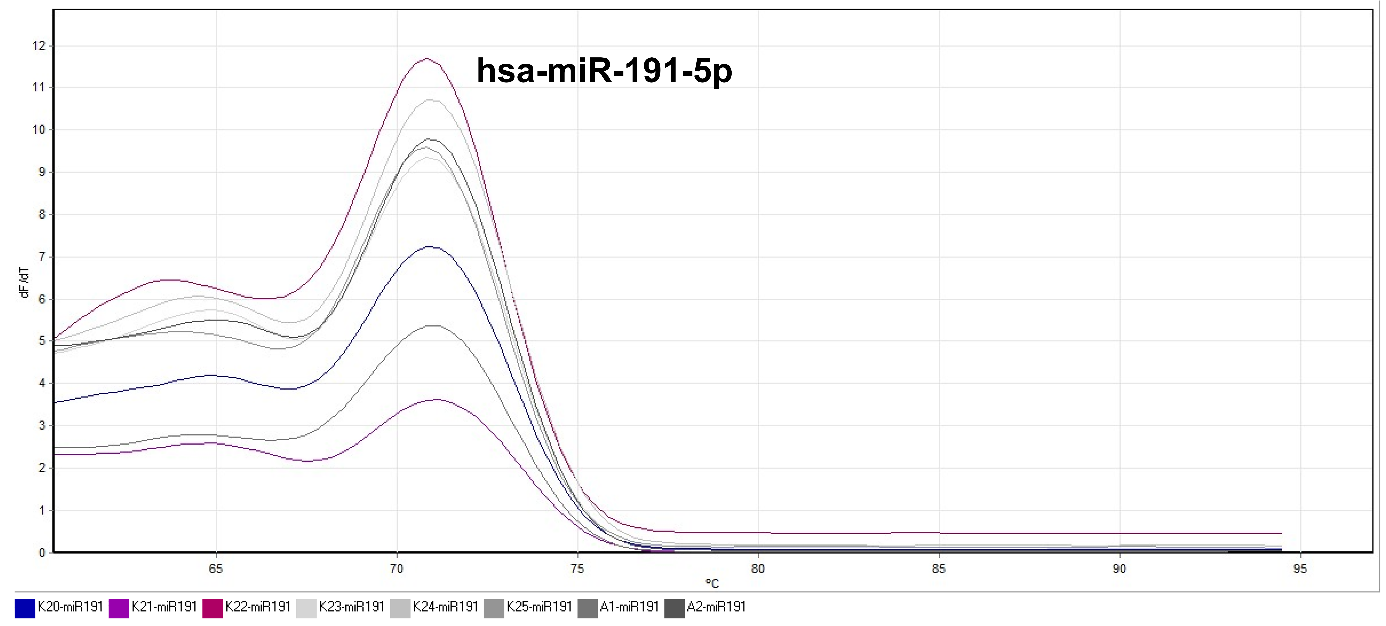


**Supplementary Fig. 4** Melting curve analysis of has-miR191-5p. Reaction condition: 1°C rise between 60-95°C

**Supplementary Fig. 5**


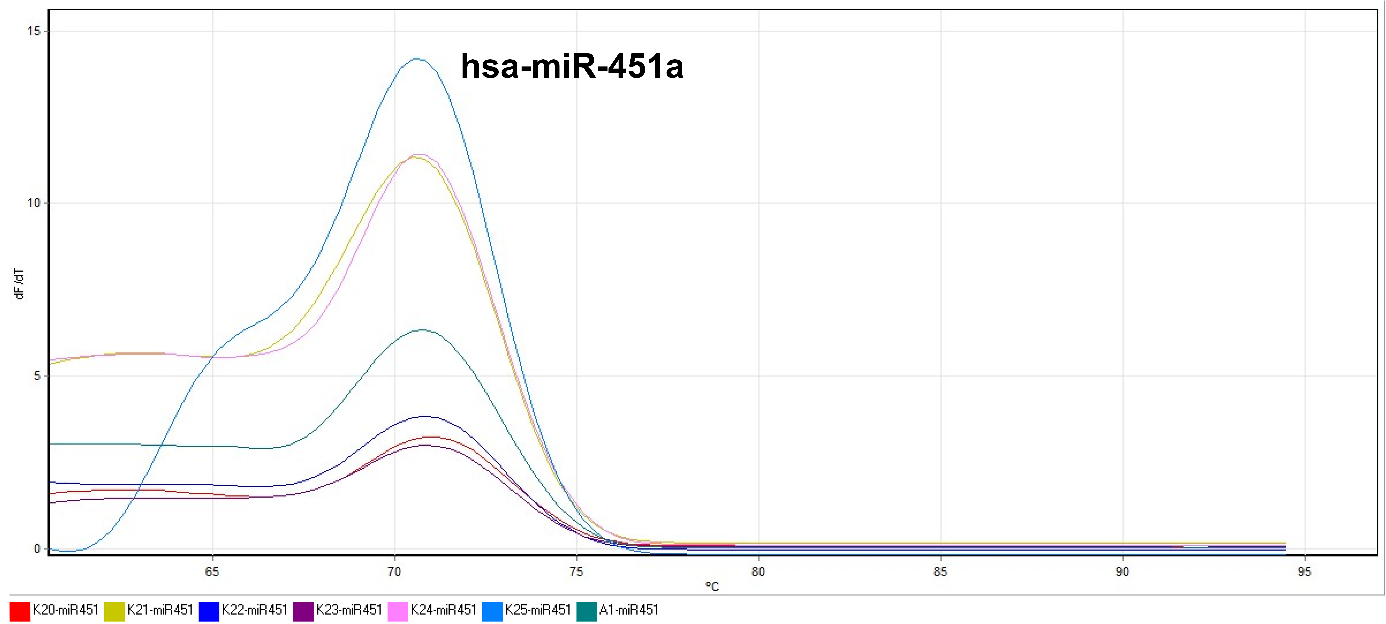


**Supplementary Fig. 5** Melting curve analysis of has-miR451a. Reaction condition: 1°C rise between 60-95°C
