# Supplementary material for: miR-451a and IL18 can differentiate familial Mediterranean fever patients in attack and remission periods: a prospective cross-sectional study
Source: Clin Rheumatol. 2025 Feb 11;44(4):1691–704. doi: 10.1007/s10067-025-07359-2 (PMC11993460; doi:10.1007/s10067-025-07359-2)
Supplement: Supplementary file 2 — Supplementary file2 (DOCX 13.7 KB) [file 10067_2025_7359_MOESM2_ESM.docx]

|  | **Cut-off** | **Sensitivity** | | **Specificity** | | **AUC (%95 CI)** | | ***p*-value** | |  |
| --- | --- | --- | --- | --- | --- | --- | --- | --- | --- | --- |
| **IL18,** **pg/mL (FMF all vs. control)** | > 224.6 | | 65.00 | | 68.00 | | 0.692 (0.58 - 0.80) | | **0.0054*** | |
| **IL18,**  **pg/mL (FMF-A vs. FMF-R)** | > 243.4 | | 70.00 | | 50.00 | | 0.689 (0.55 - 0.82) | | **0.0120*** | |
| **miR-451a (FMF all vs. control)** | < 118.2 | | 70.48 | | 48.00 | | 0.598 (0.45 - 0.75) | | 0.2141 | |
| **miR-451a (FMF-A vs. FMF-R)** | < 129.4 | | 73.33 | | 62.07 | | 0.703 (0.57 - 0.84) | | **0.0073*** | |

**Supplementary Table 1** ROC analyses of all FMF patients (*n*=60) vs. control (*n*=25) and FMF-A (*n*=30) vs. FMF-R (*n*=30) patients

*p* < 0.05 denotes statistical significance (typed in bold). ROC: Receiver operating characteristic, FMF-A, -R: Familial Mediterranean Fever-Attack, -Remission, IL18: Interleukin-18, sFAS: Soluble Fas cell surface death receptor.
